# A Task-Related EEG Microstate Clustering Algorithm Based on Spatial Patterns, Riemannian Distance, and a Deep Autoencoder

**DOI:** 10.3390/brainsci15010027

**Published:** 2024-12-29

**Authors:** Shihao Pan, Tongyuan Shen, Yongxiang Lian, Li Shi

**Affiliations:** 1 Department of Automation, Tsinghua University, Beijing 100084, China; psh19@mails.tsinghua.edu.cn (S.P.); lianyx20@mails.tsinghua.edu.cn (Y.L.); 2 School of Economics and Management, Beihang University, Beijing 100084, China; shenty@buaa.edu.cn

**Keywords:** microstate analysis, EEG clustering, spatial pattern, Riemannian distance, deep autoencoder

## Abstract

Background: The segmentation of electroencephalography (EEG) signals into a limited number of microstates is of significant importance in the field of cognitive neuroscience. Currently, the microstate analysis algorithm based on global field power has demonstrated its efficacy in clustering resting-state EEG. The task-related EEG was extensively analyzed in the field of brain–computer interfaces (BCIs); however, its primary objective is classification rather than segmentation. Methods: We propose an innovative algorithm for analyzing task-related EEG microstates based on spatial patterns, Riemannian distance, and a modified deep autoencoder. The objective of this algorithm is to achieve unsupervised segmentation and clustering of task-related EEG signals. Results: The proposed algorithm was validated through experiments conducted on simulated EEG data and two publicly available cognitive task datasets. The evaluation results and statistical tests demonstrate its robustness and efficiency in clustering task-related EEG microstates. Conclusions: The proposed unsupervised algorithm can autonomously discretize EEG signals into a finite number of microstates, thereby facilitating investigations into the temporal structures underlying cognitive processes.

## 1. Introduction

The human brain represents a complex distributed network system that can generate a wide range of neural activity patterns [1,2]. Despite the high complexity of the brain, its structural and functional connectivity limits the potential configurations of its neural activity to a lower-dimensional state space [3,4,5]. The well-established technique of microstate analysis [6] provides compelling evidence that spontaneous brain activities during the resting state can be categorized into a limited number of recurrent, quasi-stable states known as microstates. Each microstate lasts between 60 ms and 120 ms and is characterized by a distinct scalp electric field map, which represents the corresponding spatial pattern of a particular neural activity during the resting state [7,8]. Inspired by the initial research, most studies have performed microstate analyses of resting-state EEG data [9,10,11]. Recent research has attempted to extend the microstate analysis to the post-task resting-state EEG analysis to explore dynamic brain activity during memory tasks [12] and under post-training conditions [13,14]. However, performing microstate analysis on task-related EEG and segmenting cognitive processes within stable microstates remains challenging due to the rapid fluctuations of transient brain states during cognitive tasks [15,16,17].

Meanwhile, research in the field of BCIs has primarily concentrated on task-related EEG; however, the main objective has been classification, rather than the segmentation and clustering of microstates. In the field of BCIs, researchers have developed a variety of algorithms for the classification of EEG signals [18]. For instance, the common spatial pattern method [19], the filter bank common spatial pattern method [20], and a variety of spatial pattern-based algorithms are widely employed in motor imagery tasks [21,22,23,24] and emotion recognition tasks [25,26]. With the rapid advancement of deep learning, many neural network frameworks have been employed for task-related EEG classification. These technologies facilitate the automatic extraction of relevant temporal and spatial features from EEG signals, thereby markedly improving classification performance. Due to the extensive amount of the relevant literature, readers are encouraged to refer to the review articles [27,28] for a comprehensive understanding.

Although the aforementioned methods have shown promising performances in classifying task-related EEG signals, they still suffer from several limitations, including long calibration times, susceptibility to noise and outliers, and poor cross-subject transferability. Recent research demonstrates that the Riemannian approach is a highly promising tool for EEG signal classification [29,30]. This method treats the spatial covariance matrix (SCM) of EEG signals as points within the manifold of symmetric positive definite matrices. By defining affine-invariant Riemannian metrics on this manifold, a Riemannian manifold is established [31]. Given the affine invariance of the Riemannian distance within the manifold, this approach is robust to noise and outliers [32], demonstrating superior performance in transfer learning across subjects and even across multiple cognitive tasks [33,34].

In summary, current microstate analysis primarily focuses on resting-state EEG, while the field of BCIs, though concerned with task-related EEG, predominantly emphasizes the supervised or unsupervised classification [35,36] of entire EEG segments rather than clustering distinct moments within the EEG. Building on this background, the present study proposes an innovative EEG microstate clustering algorithm specifically designed for task-related EEG data. The proposed algorithm, referred to as SPADE (Spatial Patterns, Riemannian Distance, and AutoEncoder), integrates spatial patterns, Riemannian distance, and a variation of deep autoencoder for effective microstate clustering. The SPADE algorithm is designed to explore the temporal dynamics of task-related EEG data. In its implementation, spatial pattern distance and Riemannian distance are computed to quantify the variations in neural activity over time. Subsequently, a variant of the deep autoencoder is used to learn the manifold structure of these features and map them onto a low-dimensional hidden space. Finally, clustering is performed in the hidden space, and the resultant clusters are optimized iteratively.

To validate and analyze the effectiveness of the proposed SPADE algorithm, cluster analyses of simulated EEG data and of two EEG datasets related to visual tasks are performed. The experimental results indicate that the proposed SPADE algorithm is effective for generating accurate clustering results and can detect subtle changes in neural activity patterns in both simulated and real EEG data.

## 2. Materials and Methods

### 2.1. Proposed Algorithm Framework

**Figure 1:**
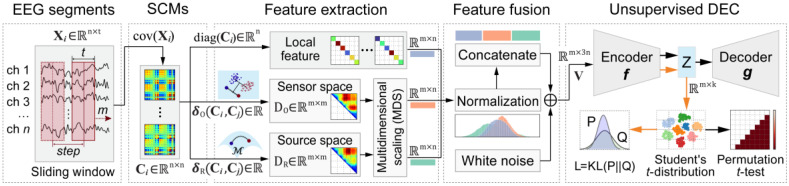
The framework of the proposed SPADE algorithm. In the first step, the total number of EEG channels is denoted by *n*, the number of data points in the sliding window is labeled by *t*, and the total number of EEG segments is marked by *m*. In the second step, the function $cov\left(\cdot \right)$ calculates the SCMs. In the third step, the function $diag\left(\cdot \right)$ converts the diagonal elements of a matrix into a vector; $\delta_{O}$ and $\delta_{R}$ represent the distances between two EEG segments in the sensor and source spaces, respectively. In the fourth step, the three features extracted from the same EEG segments are transformed and normalized before being fused into a single vector. Finally, in the fifth step, the unsupervised deep embedded clustering (DEC) algorithm is used to map the vectors to the latent space and complete the clustering process; Z represents the latent space, and k represents its dimension.

The proposed unsupervised microstate clustering algorithm, the SPADE, includes five steps, as illustrated in Figure 1. The first step involves segmenting the EEG signals into discrete timeframes using a sliding window of a length *t* and step intervals of *s*. In the second step, the SCMs of each EEG segment are computed.

The third step represents feature extraction, where the first feature to be extracted is the set of diagonal elements of SCMs. These elements represent the variance of the signal at each electrode and denote local features of the EEG segments. The second extracted feature measures the linear separability of two EEG segments. This metric is termed the spatial pattern distance in the sensor space. The third extracted feature is the geodesic distance between two SCMs of different EEG segments. This affine-invariant distance on the Riemannian manifold is unaffected by the mixing matrix, and it is referred to as the spatial pattern distance in the source space. After calculating the two spatial pattern distances between each pair of EEG segments, a multidimensional scaling algorithm is employed. This algorithm maps the relative distances between EEG segments in a high-dimensional space onto coordinates in a lower-dimensional space.

The fourth step of the proposed algorithm involves fusing three features of the same EEG segment into a single vector. First, the extracted features are transformed to ensure that they exhibit similar normal distribution characteristics. For example, in this study, the diagonal elements of the SCMs are log-transformed. Next, the three features extracted from the same EEG segments are concatenated to form a single feature vector. This vector encapsulates local features and contains global spatial pattern distances that are crucial for microstate clustering.

The fifth step of the proposed algorithm involves unsupervised deep embedding clustering (DEC) [37]. First, a deep autoencoder is trained to map the input data into a low-dimensional latent space. Then, clustering is performed in this latent space, and the Kullback–Leibler (KL) divergence between a Student’s *t*-distribution and the target distribution is used to jointly optimize the clustering centers and the latent space.

### 2.2. Detailed Algorithm Procedure

In this section, each step of the proposed SPADE algorithm will be explained in detail in the following subsections.

#### 2.2.1. Calculate Spatial Pattern Distance in the Sensor Space

Let $X_{i}\in {\mathbb{R}}^{n\times t},\;i=1,\cdots ,m$ be a multichannel EEG segment with a zero mean, where n, t, and m denote the number of electrodes, the number of data points in each sliding window, and the number of available segments, respectively. The SCM of each EEG segment can be estimated as $C_{i}=cov\left(X_{i}\right)=\frac{1}{t-1}X_{i}{X_{i}}^{T}\in {\mathbb{R}}^{n\times n}$, where ${\left(\cdot \right)}^{T}$ is the transpose operator.

Further, for each pair of EEG segments (i.e., $X_{i}$ and $X_{j}$ $\left[j=1,\cdots ,m\right]$), an optimal spatial filter $w\in {\mathbb{R}}^{n\times 1}$ is determined to maximize the ratio of the squared magnitudes of the resultant filtered signals. The objective function of w is defined as follows:

(1) $$f\left(w\right)=\frac{\left(w^{T}X_{i}\right){\left(w^{T}X_{i}\right)}^{T}}{\left(w^{T}X_{j}\right){\left(w^{T}X_{j}\right)}^{T}}=\frac{w^{T}X_{i}X_{i}^{T}w}{w^{T}X_{j}X_{j}^{T}w}=\frac{w^{T}C_{i}w}{w^{T}C_{j}w}.$$

This equation adheres to the generalized Rayleigh quotient form as $f\left(w\right)=R\left(C_{i},C_{j},w\right)$. When w corresponds to the generalized eigenvector of $C_{i}$ and $C_{j}$, function $f\left(w\right)$ reaches an extremum, which is exactly the largest generalized eigenvalue (for a detailed proof, please refer to the Appendix A).

In this study, the largest generalized eigenvalue of $C_{i}$ and $C_{j}$ is denoted by $\lambda_{max}$, and it is used as a quantitative metric for assessing dissimilarity between pairs of EEG segments. We refer to this metric as the spatial pattern distance in the sensor space:

(2) $$\delta_{O}\left(C_{i},C_{j}\right)=\arg\;\underset{w}{\max}f\left(w\right)=\lambda_{max}.$$

When two EEG segments are identical, their spatial pattern distance in the sensor space is, by definition, equal to one. When two EEG segments are not identical, we can calculate the SCMs of each segment; their spatial pattern distance is equal to the maximum generalized eigenvalue of the two SCMs.

#### 2.2.2. Calculate Spatial Pattern Distance in the Source Space

The SCMs $C_{i}\in {\mathbb{R}}^{n\times n}$ are symmetric and positive definite, and they reside on the symmetric positive definite (SPD) matrix manifold M, which represents a surface with a dimension of $n\left(n+1\right)/2$ and can be defined as follows:

(3) $$M=\left\{C_{i}\in {\mathbb{R}}^{n\times n}|C_{i}=C_{i}^{T}\;and\;\forall x\in {\mathbb{R}}^{n},\;x\neq 0,x^{T}C_{i}x>0\right\}.$$

After being equipped with the affine-invariant Riemannian metric [31] in each tangent space $T_{p}M$ of the SPD matrix manifold, a Riemannian manifold is obtained. The Riemannian distance between two SPD matrices is given by the following equation:

(4) $$\delta_{R}\left(C_{i},C_{j}\right)={\left\|logm\left(C_{i}^{-1/2}C_{j}C_{i}^{-1/2}\right)\right\|}_{F}=\sqrt{\sum_{e=1}^{N_{e}}log^{2}\lambda_{e}},$$

where $logm\left(\cdot \right)$ denotes the matrix logarithm operator, $\lambda_{e},\;e=1,\cdots ,N_{e}$ represents the eth eigenvalue of matrix $C_{i}^{-1/2}C_{j}C_{i}^{-1/2}$, and ${\left\|\cdot \right\|}_{F}$ denotes the Frobenius norm operation, which is defined as ${\left\|A\right\|}_{F}=\sqrt{{\left|A_{ij}\right|}^{2}}=\sqrt{tr\left(AA^{T}\right)}$.

Based on the definition of the Riemannian distance, the Fréchet mean (also known as the Riemannian or geometric mean) of a set of SPD matrices can be defined as the element $\bar{E}$ that minimizes the sum of squared distances $\delta_{R}$ to the points $E_{i}$ in a dataset, and it is calculated by the following equation:

(5) $$\bar{E}=\underset{E}{argmin}\sum_{i=1}^{m}\delta_{R}^{2}\left(E_{i},E\right).$$

In the Riemannian-based BCI applications, it is common to calculate the Fréchet mean of a dataset before applying the logarithm mapping to each data point relative to this Fréchet mean. This process maps a dataset onto the tangent space of the mean, which is locally homeomorphic to the Euclidean space. The logarithm mapping of point C at a given point $\bar{E}$ is expressed by the following equation:

(6) $$Log_{\bar{E}}\left(C\right)={\bar{E}}^{\frac{1}{2}}logm\left({\bar{E}}^{-\frac{1}{2}}C{\bar{E}}^{-\frac{1}{2}}\right){\bar{E}}^{\frac{1}{2}}.$$

The Riemannian distances possess an important property, termed affine invariance or congruence invariance, which can be expressed by the following equation:

(7) $$\delta_{R}\left(A,B\right)=\delta_{R}\left(MAM^{T},MBM^{T}\right),$$

where M denotes an arbitrary invertible matrix with the same size as SCMs A and B. A detailed mathematical proof of this property is discussed elsewhere [31,38,39].

In the instantaneous linear mixed model of EEG, the recorded signal is derived from a linear combination of neural source activities. Thus, the relationship between the EEG signal in the sensor space and the neural source activity in the source space can be represented by the following equation:

(8) $$X_{i}=MS_{i},$$

where M denotes the mixing matrix, and $S_{i}$ represents the neural source activity.

According to the linear mixed model, the relationship between the SCMs in the sensor and source spaces can be determined. Specifically, $C_{i}=\frac{1}{t-1}X_{i}{X_{i}}^{T}=\frac{1}{t-1}MS_{i}{S_{i}}^{T}M^{T}=MN_{i}M^{T}$, where $N_{i}$ represents the SCMs of the neural source activity.

By integrating the linkage between the SCMs in the sensor and source spaces with the congruence invariance property of Riemannian distance (as shown in Equation (7)), an important EEG signal processing property can be derived as follows:

(9) $$\delta_{R}\left(C_{i},C_{j}\right)=\delta_{R}\left(MN_{i}M^{T},MN_{j}M^{T}\right)=\delta_{R}\left(N_{i},N_{j}\right).$$

According to Equation (9), the Riemannian distance between the SCMs remains invariant during the transformation from the sensor space to the source space [30]. In this study, we therefore refer to the Riemannian distance between SCMs as the spatial pattern distance in the sensor space.

#### 2.2.3. Multidimensional Scaling and Normalization

After the spatial pattern distances in the sensor and source spaces are calculated, the two corresponding distance matrices, denoted by $D_{O}\in {\mathbb{R}}^{m\times m}$ and $D_{R}\in {\mathbb{R}}^{m\times m}$, are obtained. Each matrix element quantitatively reflects the spatial pattern discrepancy between pairs of EEG segments. Moreover, the diagonal elements of the SCM are used to represent the variance of electrode activity in individual EEG segments. These elements are denoted by $diag\left(C_{i}\right)\in {\mathbb{R}}^{n}$, where $diag\left(\cdot \right)$ is the operation that converts the diagonal elements of a matrix into a vector.

The three features collectively encapsulate the local and global characteristics of EEG segments. However, given the distinct ranges and dimensions of these features, they need to be standardized to ensure their equal contributions to the subsequent clustering analysis. To this end, the multidimensional scaling algorithm [40] is used in this study to transform an m×m spatial pattern distance matrix into m×n feature vectors. The multidimensional scaling algorithm conducts eigenvalue decomposition on the inner product of the distance matrix to determine the coordinates of points in the Euclidean space. The main objective is to preserve the pairwise distances between points as accurately as possible. Furthermore, log-normalization is applied to the diagonal elements of the SCMs. These transformations ensure that the three aforementioned features not only have uniform dimensions but also exhibit similar characteristics of a normal distribution.

#### 2.2.4. Unsupervised DEC

A variant of the deep autoencoder, which uses the unsupervised DEC algorithm framework [37], is used to facilitate the joint optimization of nonlinear embedding and clustering tasks. On benchmark tasks, the DEC exhibits state-of-the-art performance in terms of accuracy and speed and shows robustness to hyperparameter settings.

The DEC algorithm used in this study includes four steps. The first step involves training a classical autoencoder using an end-to-end encoding and decoding process to capture the underlying manifold structure of the input feature vector, denoted by $V\in {\mathbb{R}}^{m\times 3n}$, where 3*n* represents the length of the vector. Following the autoencoder training, *m k*-dimensional latent vectors are extracted from the bottleneck layer, each corresponding to an EEG segment; the latent vectors are denoted by $Z\in {\mathbb{R}}^{m\times k}$. Subsequently, the *K*-means algorithm is employed to identify *s* clustering centers in the latent space; these centers are denoted by $U\in {\mathbb{R}}^{s\times k}$.

In the second step, the DEC algorithm uses a Student’s *t*-distribution to compute the soft assignment probabilities for latent vectors $Z_{i}$ with respect to each clustering center $U_{j}$. The third step involves the construction of an auxiliary target distribution and the computation of the KL divergence between the soft assignment probabilities and auxiliary target distribution, which is then used as a clustering loss function. The final step is dedicated to minimizing the KL divergence by jointly optimizing cluster centers *U* and encoder parameters. This iterative process, which cycles from the second to the fourth step, persists until the algorithm either reaches a predefined number of iterations or the KL divergence stabilizes within a designated threshold.

### 2.3. Statistical Testing of Clustering Results

The determination of the optimal number of clusters represents a fundamental challenge in unsupervised cluster analysis [41]. Namely, excessive clustering can overcomplicate the results, making them difficult to interpret and analyze. Conversely, using fewer clusters can lead to substantial information loss, thus failing to capture the dynamic characteristics inherent in the EEG data. It has been widely accepted that in the resting state with closed eyes, four canonical microstates account for approximately 80% of the variance in EEG signals [11,15]. However, when the eyes are open and visual sensory input is available, up to 15 microstates might be required to explain a similar proportion of data variance in EEG data [16].

In this study, the clustering results are evaluated by conducting *t*-tests on the sampling distributions of Riemannian distances of elements from the same and different clusters. First, for each cluster pair, 1000 pairs of elements were sampled from each identical cluster, obtaining the sampling distribution of Riemannian distances in these clusters, which was referred to as Distribution 1. Next, 1000 pairs of elements were randomly sampled from different clusters to obtain the sampling distributions of Riemannian distances between distinct clusters, which was labeled as Distribution 2. Finally, a one-tailed *t*-test was performed on the two Riemannian distance distributions.

The clustering results were reliable when the Riemannian distances in Distribution 1 were significantly smaller than those in Distribution 2. Consequently, the statistical results of the one-tailed *t*-test were expected to be below the predefined significance threshold. Considering that multiple tests were conducted across all possible combinations of the two distinct clusters during the statistical evaluation, the resulting statistical test results were additionally adjusted using the false discovery rate (FDR) correction, with a significance threshold of *α* = 0.05.

### 2.4. Simulated EEG Data and Baseline Clustering Algorithms

**Figure 2:**
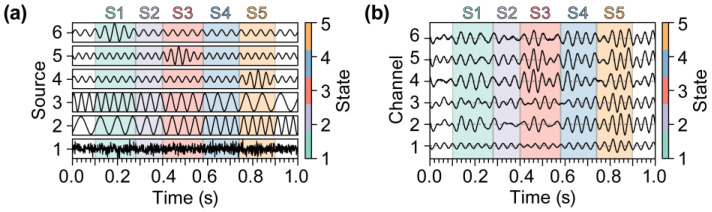
Simulated neural source activity signals and EEG signals. (**a**) Simulated neural source activity signals. These signals contained activity from six neural sources and were divided into five microstates denoted by S1–S5. The first source simulated the background noise of neural activity; the second and third sources simulated the neural source activity with a modulated frequency; the fourth, fifth, and sixth sources simulated neural source activity with the modulated amplitude in the early, middle, and late stages, respectively. (**b**) Simulated EEG signals. These EEG signals encompassed six channels and had a duration of 1000 ms and a sampling rate of 1000 Hz; they were generated by combining source neural activity signals using a random mixing matrix.

The instantaneous linear mixed model of EEG defined by Equation (8) was used to construct an artificial EEG signal to simulate a particular cognitive process. First, six unique neural source activities, each with a duration of 1000 ms and a sampling frequency of 1000 Hz, were manually designed, as illustrated in Figure 2a. Among these activities, activity 1 consists of Gaussian white noise. Activities 2 and 3 consist of chirp signals [42] with frequencies that vary linearly from 10 Hz (±5 Hz) to 30 Hz (±5 Hz) and inversely from 30 Hz (±5 Hz) to 10 Hz (±5 Hz), respectively, while maintaining a constant amplitude. Activities 4 to 6 comprise sine waves modulated by Gaussian pulses [42]. Each sine wave features a random starting phase, an amplitude of 0.5, and frequencies of 30 Hz, 20 Hz, and 10 Hz, with a random fluctuation of ±5 Hz. The Gaussian pulses have a duration of 300 ms and a maximum amplitude of 1. The center times of the pulses are set at 800 ms, 500 ms, and 200 ms, each incorporating a random fluctuation of ±50 ms. We used these activities to simulate neural activities characterized by amplitude modulation, frequency modulation, and background noise.

Then, a random mixing matrix was used to simulate the volume conduction effect and integrate these activities into a synthesized EEG signal, as shown in Figure 2b. The resulting EEG signal comprised six channels, with a duration of 1000 ms, and was sampled at 1000 Hz. Based on the frequency and amplitude characteristics of the neural source activity, the simulated EEG signal was divided into five microstates, denoted by S1–S5, as shown in Figure 2a,b. In the experiment, we used the aforementioned method to randomly generate 50 trials of EEG data and then calculated the average to obtain the final simulated EEG.

**Figure 3:**
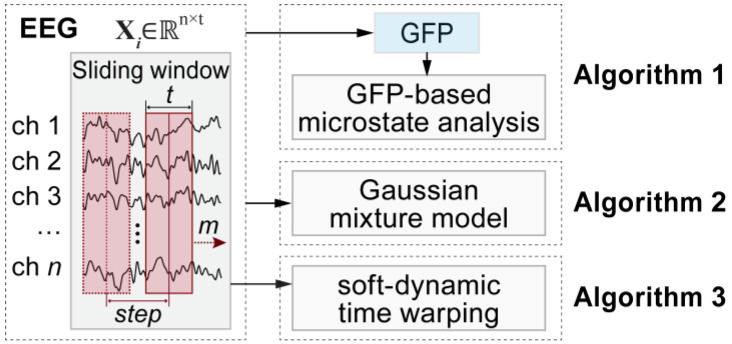
Overview of the first three baseline clustering algorithms used in this study. Algorithm 1 was the traditional global field power (GFP)-based microstate analysis method; Algorithm 2 was the Gaussian mixture model method; Algorithm 3 denoted the soft-dynamic time warping method. Algorithms 1 and 2 performed direct clustering on all time points of continuous EEG data, whereas Algorithm 3 performed clustering on EEG segments.

**Figure 4:**
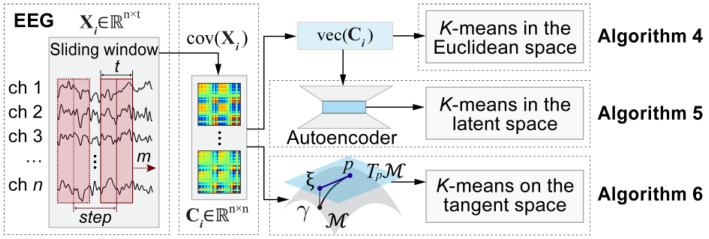
Overview of the last three baseline clustering algorithms used in this study. Algorithms 4–6 were based on the spatial covariance matrices (SCMs) of EEG segments. Algorithm 4 performed *K*-means clustering in the Euclidean space of SCMs, Algorithm 5 conducted *K*-means clustering in the latent space of an autoencoder. Algorithm 6 performed *K*-means clustering in the tangent space of the Fréchet mean of all SCMs. The function $cov\left(\cdot \right)$ computes the SCM of an EEG segment, while the function $vec\left(\cdot \right)$ converts the diagonal elements and the upper triangular elements of a SCM into a vector.

In this study, six baseline clustering algorithms were used to conduct a microstate clustering analysis of simulated EEG data. For simplicity, these algorithms are referred to as Algorithm 1, Algorithm 2, Algorithm 3, Algorithm 4, Algorithm 5, and Algorithm 6, as illustrated in Figure 3 and Figure 4.

Algorithm 1 was the global field power (GFP)-based microstate analysis method [43], which has been widely used in studies on resting-state EEG. During the calculations, the algorithm first calculated and extracted the maxima of the GFP from the simulated EEG. Next, *K*-means clustering was performed on the EEG data at the locations of the GFP peaks. Finally, the EEG data at non-peak time points were assigned to the cluster corresponding to the nearest GFP peak in time.

Algorithm 2 was the Gaussian mixture model (GMM) algorithm [44,45], which is widely used for clustering high-dimensional data. This algorithm assumes that the data are composed of a mixture of multiple normal distributions. In this paper, we treated the simulated EEG data, consisting of 6 channels and 1000 time points, as 1000 samples in a 6-dimensional space. We applied the GMM algorithm with 5 Gaussian distributions, corresponding to 5 microstates, to model these samples.

Algorithm 3 refers to the soft dynamic time warping algorithm (Soft-DTW) [46,47]. Soft-DTW is a powerful tool for comparing time series, utilizing dynamic programming to identify the optimal alignment path between two time series. The distances calculated by this algorithm are smoother and more robust than those obtained using traditional Euclidean distance, making it widely applicable in tasks such as time series classification and pattern discovery. In this paper, we started by segmenting the simulated EEG data into 800 segments using a sliding window of 200 ms and a step size of one data point. Following this, we applied the Soft-DTW algorithm to cluster these segments into five distinct clusters.

Algorithms 4–6 are based on the SCMs, which are widely used in the BCI field. The diagonal elements of the SCMs represent the variance of the data for each electrode, while the off-diagonal elements indicate the covariance between each pair of electrodes. As a second-order statistical measure, SCMs capture the intricate characteristics of EEG signals. To calculate the SCM, this study first employs a sliding window with *t *= 24 time points and *s* = 1 time point to segment the simulated EEG data into 800 segments. Subsequently, the SCM is computed for each segment. The SCM is a symmetric matrix, therefore, for an n×n SCM, the degrees of freedom are equal to $n\left(n+1\right)/2$.

Algorithm 4 begins by flattening the SCMs into one-dimensional vectors. For simulated EEG data comprising six channels (*n* = 6), the SCM is transformed into a 21-element one-dimensional vector. Then, *K*-means clustering is applied to these 800 vectors.

Similarly, Algorithm 5 initially flattens the SCMs into 21-element, one-dimensional vectors. Subsequently, an autoencoder is employed to extract features from these vectors within the latent space. Ultimately, *K*-means clustering is conducted on the resulting 800 feature vectors in this latent space. For a detailed overview of the autoencoder’s structure, please refer to Table A1 in Appendix B.

In contrast, Algorithm 6 [48] does not flatten the SCMs into vectors. Instead, it calculates the Fréchet mean of the 800 SCMs as defined by Equation (5). It then applies a logarithmic mapping to all SCMs, as defined by Equation (6), to map them to the tangent space of the Fréchet mean. Finally, *K*-means clustering is performed on the tangent space of the Fréchet mean.

Despite significant advancements in current deep learning algorithms, most of these primarily focus on supervised or semi-supervised learning tasks, with relatively few algorithms tailored for unsupervised clustering tasks. Furthermore, EEG data collected in cognitive studies are often highly limited, which can render complex deep learning algorithms less suitable for EEG clustering. Although some researchers have proposed clustering algorithms specifically for EEG, these trial-based clustering algorithms [35,36] primarily focus on clustering entire EEG segments. In contrast, our proposed SPADE algorithm clusters distinct moments within a segment of EEG. Therefore, we limited the baseline algorithms to the six mentioned above.

### 2.5. Real EEG Datasets and Preprocessing

Two publicly available real EEG datasets [49] are utilized to evaluate the effectiveness of the proposed microstate clustering algorithm. The first dataset is derived from a face perception experiment, while the second dataset is from a word-pair judgment experiment. The face perception experiment focuses on studying the differences in brain activity related to the perception and categorization of basic visual stimuli, whereas the word-pair judgment experiment examines the differences in brain activity associated with higher-level semantic processing. These two scenarios encompass a broad spectrum of brain function, ranging from basic visual perception to higher-level semantic cognition. By using these two datasets, we aim to demonstrate the versatility and robustness of the proposed SPADE algorithm. If the algorithm can successfully distinguish the subtle differences in brain activity during these representative tasks, it has the potential to be applied to a wider range of cognitive research. This comprehensive evaluation ensures that the algorithm’s performance is not limited to a specific type of cognitive task and can be generalized to various brain functions.

In the face perception task, at the 0 ms mark of each trial, either a face, a car, a scrambled face, or a scrambled car was presented at the center of the screen for 300 ms. This was followed by a blank screen that lasted for a random duration between 1100 and 1130 ms. During this interval, participants were required to indicate whether the presented image was scrambled. In the word-pair judgment task, each trial began with the presentation of a red prime word at the center of the screen for 200 ms, followed by a blank screen that lasted for a random duration between 900 and 1100 ms. Subsequently, the 0 ms mark was established, and a green target word was displayed at the center of the screen for 200 ms. This was followed by another blank screen that appeared for a random duration between 1100 and 1300 ms. During this final interval, participants were instructed to indicate whether the target word was semantically related or unrelated to the prime word.

**Figure 5:**
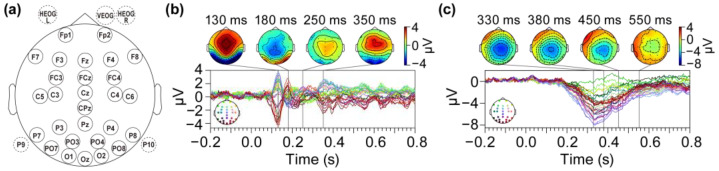
Grand-average EEG difference waves between conditions in two datasets. (**a**) The montage of the 33 electrodes. The five electrodes indicated by the dashed circles represent those that were ultimately excluded. The final two EEG datasets comprised 28 channels, with a time range of −200 to 800 ms and a sampling rate of 256 Hz. (**b**) Difference waves of the grand-average EEG responses between the face and car stimuli conditions in Dataset 1. (**c**) Difference waves of the grand-average EEG responses between semantically unrelated and related target words in Dataset 2.

Forty participants (25 females and 15 males; mean age = 21.5, SD = 2.87, and range 18–30) took part in two dataset-related experiments. Continuous EEG was recorded from 33 scalp electrodes placed according to the International 10/20 System. The montage of the 33 electrodes is illustrated in Figure 5a. All original EEG data are publicly available (accessed on 26 December 2024 at https://doi.org/10.18115/D5JW4R). The EEGLAB toolbox (version 13_4_4b) [50] was utilized for signal processing. During processing, two datasets were down sampled from 1024 Hz to 256 Hz to increase subsequent data processing speeds. Eyeblinks and horizontal eye movements were computed as VEOG minus FP2 and as left HEOG minus right HEOG, respectively. The two datasets were then re-referenced to an average reference, excluding the bipolar HEOG and VEOG channels. To ensure the full rank of the data and avoid rank-deficiency issues, we included the initial reference channels when computing the average for re-referencing. Next, DC offsets were removed, and the signals were band-pass filtered using a non-causal finite impulse response filter implemented in EEGLAB, with a frequency range of 0.1 to 40 Hz. The filter was configured with default parameters, including a filter order of 1000, a ripple of 0.1 dB, and a transition band of 0.1 Hz. Independent component analysis (ICA) was conducted using the Infomax ICA algorithm, excluding the bipolar HEOG and VEOG channels. A total of 30 components were extracted, and two components associated with eyeblinks or horizontal eye movements were removed based on visual inspection of the waveforms and scalp distributions. Finally, the bipolar HEOG, VEOG, P9, and P10 channels were discarded, resulting in a total of 28 channels remaining.

In the final step, two types of trials were selected from Dataset 1, each associated with visual stimuli of cars and faces. The epochs for these two trial types were segmented from −200 ms to 800 ms relative to the onset of the visual stimulus (either a face or a car), and baseline correction was applied using the average amplitude from −200 ms to 0 ms. In Dataset 2, all trials were categorized into semantically related and unrelated conditions, with epochs segmented from −200 ms to 800 ms relative to the onset of the target word stimulus, and baseline correction was similarly applied using the average amplitude from −200 ms to 0 ms. Finally, epochs in both datasets with amplitudes exceeding ±100 μV were excluded.

Figure 5b illustrates the difference wave of the grand-average EEG for trials corresponding to the face and car stimuli conditions. Previous research [51] has indicated that EEG signals exhibit a distinct pattern during the visual processing of faces versus non-face objects, such as cars. Specifically, a negative deflection known as the N170 component can be observed approximately 130 to 200 ms after the presentation of visual stimuli. This event-related potential can be predominantly detected over the occipitotemporal regions of the scalp. Figure 5c depicts the difference wave of the grand-average EEG for trials corresponding to the relevant and irrelevant word conditions. Previous research [52] has demonstrated that when a pair of prime and target words are sequentially presented to subjects, a semantically unrelated target word elicits a measurable negative voltage shift in electrodes positioned near the parieto-occipital or centro-parietal regions. This shift typically occurs approximately 380 milliseconds post-stimulus and is termed the N400 component.

When conducting analysis using the proposed SPADE algorithm, we first calculated the mean EEG voltage at both the individual and group levels. We then employed a sliding window with *t* = 100 ms and *s* = 1 time point, sliding from 0 ms to 700 ms, to segment each mean EEG into 180 segments. Finally, we computed the SCM for all segments and proceeded with the remaining processes as described in Figure 1.

### 2.6. Evaluation Methodology

Five evaluation criteria—Adjusted Rand Index (ARI), Normalized Mutual Information (NMI), Purity Score, F1 Score, and Silhouette score—are utilized to measure the performance of the EEG clustering algorithms. The first four evaluation metrics require the use of a ground truth as a reference, whereas the fifth metric assesses the clustering results solely based on the distances between elements.

The Adjusted Rand Index (ARI) [53] is a statistical measure used to evaluate the similarity between two data clusterings. It adjusts the Rand Index (RI) to provide a more accurate assessment of clustering quality. To calculate the RI, a confusion matrix is created based on the number of pairs of data points that are in the same or different clusters in both the true and resulting clusterings. The formula for the RI is then given by $RI=\left(TP+TN\right)/\left(TP+TN+FP+FN\right)$, where TP stands for the number of true positives; TN, true negatives; FP, false positives; and FN defines the number of false negatives. Finally, $ARI=\left(RI-E\left(RI\right)\right)/\left(max\left(RI\right)-E\left(RI\right)\right)$, where $E\left(RI\right)$ stands for the expected RI and $max\left(RI\right)$ stands for the maximum RI. The ARI ranges from −1 to 1, where 1 indicates perfect agreement.

Normalized Mutual Information (NMI) [54] is a measure of the similarity between two clusterings of the same data points, with values ranging from 0 (no mutual information) to 1 (perfect agreement). The NMI between two clusterings *U* and *V* is defined as follows:

(10) $$NMI\left(U,\;V\right)=\frac{I\left(U,\;V\right)}{\sqrt{H\left(U\right)\cdot H\left(V\right)}},$$

where $MI\left(U,\;V\right)$ is the Mutual Information between clusterings *U* and *V*. $H\left(U\right)$ is the entropy of clustering *U*. $H\left(V\right)$ is the entropy of clustering *V*.

The Purity Score quantifies the degree to which clusters predominantly consist of samples from the same class. A higher Purity Score signifies a more effective clustering result and can be calculated as follows:

(11) $$Purity\left(R,L\right)=\frac{1}{n}\underset{k}{\sum }max\left|r_{k}\cap l_{j}\right|,$$

where $R=\left\{r_{1},r_{2},\cdots ,r_{k}\right\}$ represents the clustering results, with each $r_{k}$ being a cluster; $L=\left\{l_{1},l_{2},\cdots ,l_{j}\right\}$ represents the true labels, with each $l_{j}$ being a category; *n* is the total number of data points; and $r_{k}\cap l_{j}$ represents the number of points that belong to both cluster $r_{k}$ and category $l_{j}$.

The F1 Score is the harmonic mean of precision and recall, offering a balance between the two metrics and serving as a robust measure of a model’s accuracy. $F1=2\left(P\times R\right)/\left(P+R\right)$, where $P=TP/\left(TP+FP\right)$ and $R=TP/\left(TP+FN\right)$.

Lastly, the Silhouette score [55] assesses how well each data point is clustered by considering both the cohesion within clusters and the separation between clusters. The score ranges from −1 to 1, with a higher score indicating better-defined clusters. The Silhouette score is defined as follows:

(12) $$S=\frac{1}{n}\overset{n}{\underset{i=1}{\sum }}\frac{b\left(i\right)-a\left(i\right)}{max\left(a\left(i\right),b\left(i\right)\right)},$$

where *n* is the total number of samples, $a\left(i\right)$ is the average distance of sample i to all other samples in the same cluster, and $b\left(i\right)$ is the average distance of sample i to all samples in the nearest cluster.

The first four of the five evaluation criteria depend on ground truth labels, while the fifth criterion is based on the distances between data points. Therefore, we computed all five metrics using the clustering results derived from the simulated EEG dataset, which contains ground truth labels, and exclusively calculated the fifth metric based on the clustering outcomes from two real EEG datasets. The distance employed here is the Riemannian distance between each pair of SCMs, as defined by Equation (4).

For each metric, after obtaining a set of scores for the clustering results across all data within a single dataset and across different algorithms, we conducted paired tests to compare the scores of each baseline algorithm with those of the proposed algorithm. Specifically, we initially computed the scores based on the clustering results from all algorithms. Subsequently, we performed the Shapiro–Wilk normality test [56] to determine whether these scores followed a normal distribution. If all scores conformed to a normal distribution, we conducted paired *t*-tests with FDR correction to compare the scores of the baseline algorithms with those of the proposed SPADE algorithm. Otherwise, we executed paired Wilcoxon signed-rank tests [56] with FDR correction.

## 3. Results

### 3.1. Microstate Analysis Based on Simulated EEG Data

**Figure 6:**
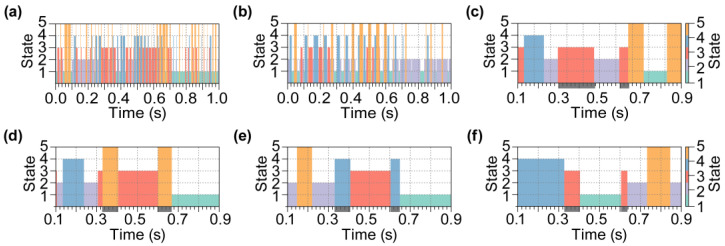
The microstate analysis results of simulated EEG data obtained by six baseline algorithms. The simulated EEG data were partitioned into a series of microstates based on the clustering results of (**a**) Algorithms 1; (**b**) Algorithms 2; (**c**) Algorithms 3; (**d**) Algorithms 4; (**e**) Algorithms 5; and (**f**) Algorithms 6. Each block, differentiated by color and height, symbolizes a unique microstate. The state indices were randomly generated during the clustering process. The gray area on the *x*-axis in subfigures (**c**–**f**) indicates the prevalent error in the microstate sequences obtained by Algorithms 3–6, respectively; these algorithms failed to segment the time period into two distinct microstates.

We first conducted clustering analysis on simulated EEG data using six baseline algorithms. During the analysis, Algorithms 1 and 2 directly partitioned the continuous simulated EEG data into five distinct clusters. In contrast, Algorithm 3 first segmented the EEG data using a sliding window with *t* = 100 ms and *s* = 1 time point. The EEG segments were categorized into five distinct clusters. In Algorithms 4–6, after segmenting the EEG data into 200 ms fragments, the SCM of each fragment was computed. Subsequently, *K*-means clustering analysis was conducted to obtain five clusters, which were used in the Euclidean space of the SCM, the latent space of the autoencoder, and the tangent space of the Riemannian manifold. After clustering, the time-series data were segmented into a sequence of microstates corresponding to the clustering results. Figure 6 presents the derived microstate sequences obtained by six baseline clustering algorithms.

**Figure 7:**
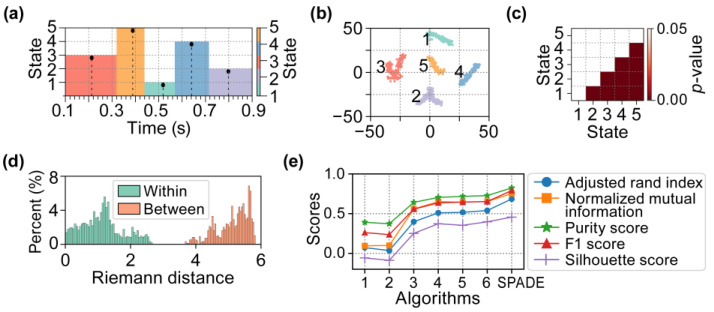
The microstate analysis results of simulated EEG data obtained by the proposed SPADE algorithm. (**a**) The simulated EEG data partitioned into a series of microstates based on the clustering results. (**b**) *t*-Distributed Stochastic Neighbor Embedding (*t*-SNE) visualization of clustering results. The numbers 1–5 represent randomly generated cluster labels. (**c**) The *t*-test results of Riemannian distances within and between each pair of clusters (FDR corrected). (**d**) The sampling distributions of Riemannian distances within (mean [M] = 1.16, standard deviation [SD] = 0.62) and between (M = 5.10, SD = 0.47) Clusters 1 and 2. (**e**) Mean scores from 30 clustering evaluations of six baseline algorithms and the proposed SPADE algorithm using five different evaluation metrics.

Subsequently, we employed the SPADE algorithm to perform a microstate clustering analysis on the simulated EEG data. The sliding window used in the computation was set to a length of 200 ms, with a step size of one time point. The architecture of the autoencoder utilized is detailed in Table A2. The resulting microstate sequences are depicted in Figure 7a, while the corresponding clusters for each microstate are visualized in Figure 7b. To demonstrate the significant differences between the identified clusters, we conducted statistical tests on the Riemann distances of both intra-cluster and inter-cluster elements for any pair of clusters. The results of these *t*-tests are presented in Figure 7c. Specifically, Figure 7d illustrates the distribution of Riemann distances for intra-cluster and inter-cluster elements in Clusters 1 and 2.

Furthermore, to evaluate the clustering accuracy of the proposed SPADE algorithm in comparison with six baseline algorithms, we generated 30 simulated EEG datasets using the method outlined in Figure 2. Each dataset was derived by averaging 50 trials, with the neural source activity parameters varying randomly across these trials, while maintaining a consistent mixing matrix. We then employed each of the seven algorithms to cluster the 30 simulated EEG datasets and calculated the five evaluation metrics mentioned in Section 2.6 for the clustering results. The mean scores of the five metrics for all seven algorithms are presented in Figure 7e, with their specific values displayed in Table 1. The results indicate that the proposed SPADE algorithm outperformed the other six baseline algorithms across all metrics.

**Figure 8:**
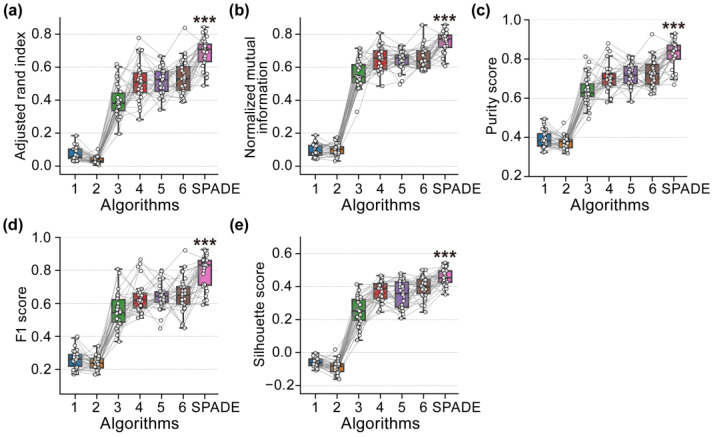
Scores and statistical results of five metrics for 30 simulated EEG clustering results from six baseline algorithms and the proposed SPADE algorithm. *** indicates that the results of the paired Wilcoxon signed-rank tests conducted between the proposed algorithm and the six baseline algorithms are all very significant (*** *p* < 0.001, FDR corrected). (**a**) Adjusted Rand Index scores and statistical results. (**b**) Normalized Mutual Information scores and statistical results. (**c**) Purity Scores and statistical results. (**d**) F1 Scores and statistical results. (**e**) Silhouette scores and statistical results.

We further conducted paired Wilcoxon signed-rank tests (with FDR correction) on the scores of five metrics for the clustering results obtained from the six baseline algorithms and the proposed SPADE algorithm across the 30 simulated EEG datasets. Statistical analysis results indicate that the proposed SPADE algorithm demonstrates an extremely significant difference in scores across each metric compared to the other six baseline algorithms. Figure 8 presents the statistical analysis results of the scores for the five metrics (*** *p* < 0.001, FDR corrected).

### 3.2. Microstate Analysis Based on EEG Data from Face and Car Perception Tasks

To validate the efficiency of the proposed microstate clustering algorithm on experimental EEG datasets, we conducted clustering analyses of EEG data from 40 subjects engaged in cognitive tasks involving the perception of faces and cars [49]. During clustering, the epochs corresponding to the two visual stimuli (i.e., cars and faces) were first averaged at the subject level and then at the group level. Subsequently, the proposed SPADE algorithm was applied to both the subject-average and grand-average EEG signals. The sliding window, with *t* = 24 time points and *s* = 1 time point, ranged from 0 ms to 700 ms within the epochs. The architecture of the autoencoder utilized is detailed in Table A3 of Appendix B. The number of clusters was set to eight and treated as a hyperparameter.

**Figure 9:**
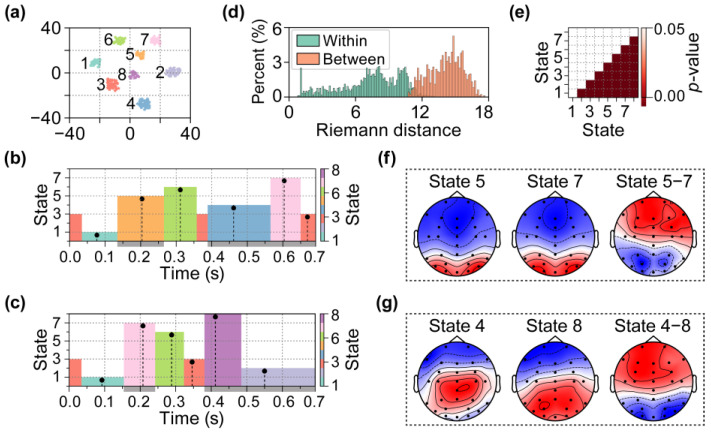
The microstate analysis of EEG data from the face and car perception tasks. (**a**) *t*-SNE visualization of clustering results. The numbers 1–8 represent randomly generated cluster labels. (**b**) The microstate series during the perception of faces. (**c**) The microstate series during the perception of cars. Each block represents a microstate corresponding to a cluster in the clustering results. The gray area on the *x*-axis denotes the time range during which the microstate sequences differed under the two conditions. The vertical black dashed line in each block indicates the Fréchet mean of Riemannian distance of the SCMs in the current cluster. The indices of the microstates were randomly generated. (**d**) The sampling distributions of Riemannian distances within (M = 7.38, SD = 2.76) and between (M = 14.15, SD = 1.49) Clusters 1 and 2. (**e**) The one-tailed *t*-test results of Riemannian distances within and between each pair of clusters (FDR corrected). (**f**) The scalp topographies of Microstates 5 and 7 and their difference. (**g**) The scalp topographies of Microstates 4 and 8 and their difference.

Figure 9a presents the *t*-Distributed Stochastic Neighbor Embedding (*t*-SNE) visualization of the clustering results. Based on these results, the EEG data were segmented into a series of microstates. Next, the Fréchet mean of the Riemannian distances of SCMs was calculated within each cluster, and the EEG data at the central time point corresponding to the Fréchet mean were considered as representative EEG data for that microstate. The results of the microstate series during the perception of faces and cars are illustrated in Figure 9b,c. Finally, statistical analyses of Riemannian distances within and between different clusters were performed to validate the clustering results. Figure 9d illustrates the sampling distributions of Riemannian distances both within and between Clusters 1 and 2; Figure 8e shows the one-tailed *t*-test results of Riemannian distances within and between each pair of clusters (FDR corrected).

The proposed SPADE algorithm could successfully delineate the distinctions in visual processing between the faces and cars. Specifically, the proposed algorithm segregated the EEG signals corresponding to the face and car stimuli within the post-stimulus interval of 150 to 250 ms and assigned them to Microstates 5 and 7, respectively. Figure 9f presents the scalp topographies of Microstates 5 and 7 and a comparative scalp topography that highlights the differences between the two microstates. At approximately 400 ms, differences in the microstate series re-emerged. Figure 9g illustrates Microstates 4 and 8 at this time, along with the differences between them.

### 3.3. Microstate Analysis Based on EEG Data from Word-Pair Judgment Task

**Figure 10:**
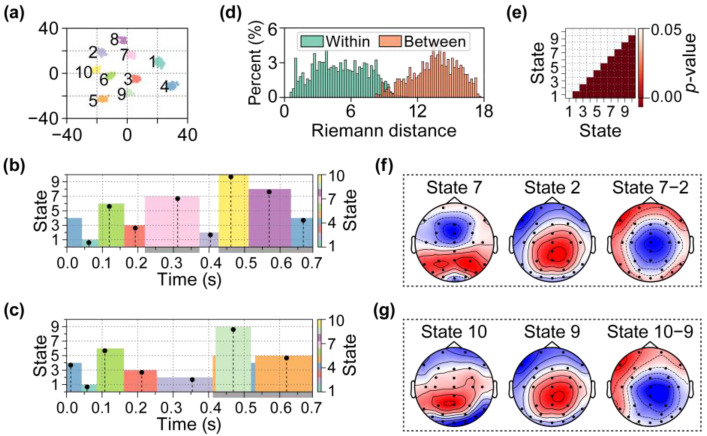
The microstate analysis of the EEG data from the word-pair judgment task. (**a**) *t*-SNE visualization of clustering results. The numbers 1–10 represent randomly generated cluster labels. (**b**) The microstate series corresponding to the condition in which the target words were semantically unrelated to the prime words. (**c**) The microstate series corresponding to the condition in which the target words were semantically related to the prime words. The gray area on the *x*-axis represents the time range during which the microstate sequences differed under the two conditions. Each block represents a microstate corresponding to a cluster in the clustering results. The vertical black dashed line in each block indicates the Fréchet mean of the SCMs in the current cluster. The indices of the microstates were randomly generated. (**d**) The sampling distributions of Riemannian distances both within (M = 6.43, SD = 3.17) and between (M = 20.43, SD = 2.39) Clusters 1 and 2. (**e**) The one-tailed *t*-test results of Riemannian distances within and between each pair of clusters (FDR corrected). (**f**) The scalp topographies of Microstates 7 and 2 and their difference. (**g**) The scalp topographies of Microstates 10 and 9 and their difference.

In the subsequent analysis, the proposed SPADE algorithm was performed on the EEG data collected during a word-pair judgment task [49]. These data encompassed EEG recordings from 40 participants engaged in discerning target words that might be semantically related or unrelated to prime words presented earlier. We employed the same parameters and processing steps as those described in the previous section to conduct microstate clustering analysis on the epochs data under both conditions. The number of clusters was set to 10. Figure 10a presents the *t*-SNE visualization of the clustering results. Based on these results, the cognitive processes under the two conditions were segmented into two series of microstates, as shown in Figure 10b,c. Figure 10d illustrates the sampling distributions of Riemannian distances both within and between Clusters 1 and 2, while Figure 10e displays the one-tailed *t*-test results of Riemannian distances within and between each pair of clusters (FDR corrected).

The microstate series presented in Figure 10b,c indicates that around 300 ms, an additional microstate, Microstate 7, emerged in the condition corresponding to semantically unrelated word pairs. Figure 10f illustrates Microstates 7 and 2 along with their differences. After 400 ms, the differences in microstates re-emerged. Figure 10g depicts Microstates 10 and 9, highlighting the variations between them.

### 3.4. Statistical Comparison of Algorithm Performance on the Two Real EEG Datasets

To further quantify the performance of the proposed SPADE algorithm on real EEG data, we conducted subject-level microstate analysis on the aforementioned two EEG datasets using baseline Algorithm 5, baseline Algorithm 6, and the SPADE algorithm. The clustering results of the three algorithms were evaluated using the Silhouette score metric across 40 participants.

During clustering, we first applied a sliding window, with *t* = 24 time points and *s* = 1 time point, to segment the epoch data from 0 ms to 700 ms under both conditions. Subsequently, we calculated the SCM for each segment. Finally, Algorithm 5 mapped the SCMs to a low-dimensional latent space for *K*-means clustering. The autoencoder used in Algorithm 5 is detailed in Table A4 of Appendix B. In contrast, Algorithm 6 mapped the SCMs to the tangent space at the same point on the manifold using Equation (6) and performed *K*-means clustering within the tangent space.

**Figure 11:**
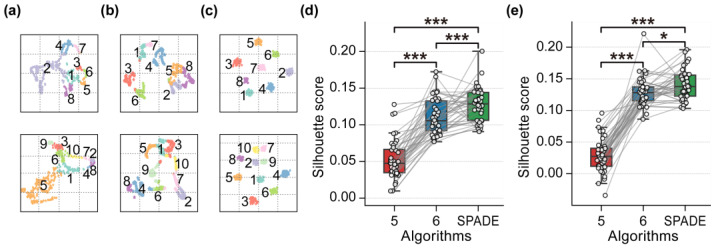
Subject-level microstate analysis results using Algorithm 5, Algorithm 6, and the proposed SPADE algorithm. (**a**) *t*-SNE visualization of clustering analysis results for EEG data of Subject 1 from Dataset 1 (upper) and Dataset 2 (lower) using Algorithm 5. (**b**) *t*-SNE visualization of clustering analysis results for EEG data of Subject 1 from Dataset 1 (upper) and Dataset 2 (lower) using Algorithm 6. (**c**) *t*-SNE visualization of clustering analysis results for EEG data of Subject 1 from Dataset 1 (upper) and Dataset 2 (lower) using the proposed SPADE algorithm. The numbers 1–10 in subfigures a–c represent randomly generated cluster labels. (**d**) Silhouette scores of clustering results for 40 subjects in Dataset 1 across three algorithms with statistical test results (FDR corrected, *** *p* < 0.001). (**e**) Silhouette scores of clustering results for 40 subjects in Dataset 2 across three algorithms with statistical test results (FDR corrected, *** *p* < 0.001, and * *p* < 0.05).

Figure 11a presents the *t*-SNE visualization of the clustering analysis results for the EEG data of Subject 1 in both Dataset 1 and Dataset 2, obtained using Algorithm 5. Figure 11b displays the corresponding results for Algorithm 6, while Figure 11c shows the results for the proposed SPADE algorithm.

After clustering, the Silhouette scores for the results obtained from three algorithms were calculated for 40 subjects in both datasets. Subsequently, Wilcoxon signed-rank tests with FDR correction were conducted for each pair of the three algorithms across the two datasets. Figure 11d depicts the Silhouette scores of the clustering results for the 40 subjects in Dataset 1 across the three algorithms, along with the outcomes of the statistical tests. Figure 11e displays the Silhouette scores of the clustering results for the 40 subjects in Dataset 2 under the three algorithms, accompanied by the results of the statistical tests. Table 2 presents the mean and standard deviation of Silhouette scores for the clustering results obtained from subject-level EEG data, based on two baseline algorithms and the proposed SPADE algorithm.

## 4. Discussion

We conducted three experiments to evaluate the performance of the proposed SPADE algorithm. In the first experiment, we initially generated simulated EEG data and subsequently compared the SPADE algorithm with six baseline algorithms using this simulated data. The comparison was conducted across five distinct scoring metrics. In the second experiment, we utilized two publicly available EEG datasets to test the SPADE algorithm’s capability in detecting subtle differences in cognitive processes. In the third experiment, we performed subject-level microstate clustering using the proposed SPADE algorithm, alongside two other excellent baseline algorithms, on these two EEG datasets. Subsequently, we compared the performance of the algorithms through statistical analysis of the Silhouette scores.

As depicted in Figure 6e, the analysis of simulated EEG data revealed that the proposed SPADE algorithm surpassed six baseline methods across all five scoring metrics, indicating its superior ability to capture the underlying microstates of the simulated EEG. Notably, Algorithms 4–6 achieved scores similar to, but not quite as high as, the proposed SPADE algorithm. These three algorithms are designed based on SCM features, which have been proven to be highly effective EEG features in the field of BCIs [30,57,58].

By comparing the clustering results of Algorithms 4–6 with the ground truth, we observed that these algorithms are unable to differentiate between State 2 and State 4, as depicted in Figure 2a. To highlight the common errors of these algorithms, we marked these areas on the x-axis in Figure 6d–f using a gray color. In the context of the simulated EEG, the distinction between State 2 and State 4 lies in the frequencies of Channel 2 and Channel 3. If Channel 2 and Channel 3 are interchangeable, then State 2 and State 4 become virtually identical. Therefore, we believe that during the process of unfolding the SCM into a one-dimensional vector, structural information among the data elements in the matrix is lost, leading to the equivalence of Channel 2 and Channel 3. With a large amount of labeled data and sufficient supervised training, the algorithms might recover this structural information from the vector data. However, the microstate clustering process of EEG involves unsupervised learning with relatively limited data, making it challenging for the algorithms to capture the structural information from the vectors. The enhanced precision of the proposed SPADE algorithm can be attributed to the extraction of holistic structural information from the SCM through the utilization of handcrafted features such as spatial patterns and Riemannian distances. This approach effectively preserves the global spatial information inherent in the data structure of the SCM.

When applied to real EEG data, the results of two cognitive tasks indicated that the proposed SPADE algorithm is suitable for the microstate analysis of task-related EEG data. The algorithm not only successfully identifies the microstate differences corresponding to the N170 and N400 components but also offers deeper insights into the brain dynamics associated with these tasks. For instance, the microstate analysis revealed that during the face and car perception tasks, the appearance of the N170 component [51] around 200 ms correlated with the brain transitioning into two distinct microstates: Microstate 5 (depicted in Figure 9b) and Microstate 7 (depicted in Figure 9c), which were linked to the perception of faces and cars, respectively. Conversely, in the word-pair judgment task, the emergence of the N400 component [52] around 380 ms was attributed to the brain transitioning into an additional microstate—specifically, Microstate 7 in Figure 10b—when encountering semantically unrelated target words.

Further analysis was conducted at the subject level using the proposed SPADE algorithm, alongside Algorithms 5 and 6, which were among the top performers in previous studies, on the two aforementioned datasets. The clustering results were evaluated using Silhouette scores. The results of the Wilcoxon signed-rank tests conducted on the Silhouette scores revealed that the proposed SPADE algorithm exhibited significant improvements in subject-level analysis. The *t*-SNE visualizations of the EEG clustering results for Subject 1, depicted in Figure 11a–c, provide intuitive insights into the performance of the algorithms. Specifically, the proposed SPADE algorithm is able to delineate clusters with clear boundaries. These results suggest that our algorithm not only captures the microstates with greater accuracy but also demonstrates robustness across individual subjects.

Additionally, compared to Algorithm 5, the autoencoder utilized in our algorithm features a decreased number of nodes in its input layer. Specifically, our algorithm constructs feature vectors of length 3*n*, whereas Algorithm 5 directly unfolds the SCM into a one-dimensional vector for input into the autoencoder, yielding a vector of length $n\left(n+1\right)/2$. The relationship between the two approaches and the number of EEG channels are linear and quadratic, respectively. Thus, as the number of EEG channels increases, the autoencoder employed in our algorithm maintains a simpler structure and fewer parameters, which is particularly beneficial in cognitive research where EEG data are often limited.

## 5. Conclusions

In this study, we introduced a novel task-related EEG microstate clustering algorithm named SPADE, which is based on spatial patterns, Riemannian distance, and a deep autoencoder. We conducted experiments on simulated EEG data and two publicly available real EEG datasets to assess the performance of the SPADE algorithm, along with six baseline algorithms. Additionally, we performed statistical analysis on the Silhouette scores of the microstate clustering results at the subject level. The experimental results demonstrate that the SPADE algorithm can precisely cluster simulated EEG data into microstates and efficiently discern subtle variations in microstates that are associated with diverse cognitive processes.

The proposed EEG microstate clustering algorithm has two important application prospects. First, the proposed algorithm could be used to partition continuous task-related EEG data into distinct microstates, thus facilitating the exploration of the temporal architecture underlying cognitive processes, which might substantially enrich the insights into brain dynamics. Secondly, the proposed algorithm employs an unsupervised technique to automatically segment EEG signals into microstate sequences. Consequently, it has the potential to advance BCI technologies by enabling the precise identification of specific microstates or microstate sequences that are characteristic of particular cognitive activities.

## Figures and Tables

**Table 1:** Mean scores of five metrics for the clustering results of 30 simulated EEG datasets using six baseline algorithms and the proposed SPADE algorithm.

Algorithm	ARI	NMI	Purity Score	F1 Score	Silhouette Score
1	0.0721	0.0946	0.3913	0.2616	−0.0575
2	0.0357	0.0995	0.3726	0.2361	−0.0876
3	0.3982	0.5591	0.6422	0.5569	0.2512
4	0.5097	0.6508	0.7043	0.6351	0.3732
5	0.5171	0.6446	0.7176	0.6459	0.3533
6	0.5393	0.6528	0.7271	0.6521	0.3974
SPADE	0.6852	0.7538	0.8241	0.7912	0.4565

**Table 2:** Mean and standard deviation of Silhouette scores for subject-level clustering results based on two baseline algorithms and the proposed SPADE algorithm.

Dataset	Algorithm 5 (Mean)	Algorithm 5 (Std)	Algorithm 6 (Mean)	Algorithm 6 (Std)	SPADE (Mean)	SPADE (Std)
1	0.0533	0.0236	0.1128	0.0275	0.1353	0.0284
2	0.0414	0.0254	0.1369	0.0125	0.1403	0.0255

## Data Availability

The data and the source code presented in this study are openly available in GitHub at https://github.com/Panshihao/SPADE.

## Appendix A

To determine the maximum value of the objective function f with respect to w.

(A1) $$f\left(w\right)=\frac{w^{T}C_{i}w}{w^{T}C_{j}w}.$$

Since both the numerator and denominator of $f\left(w\right)$ contain quadratic terms of w, the value of $f\left(w\right)$ is independent of the magnitude of vector w and depends solely on the direction of vector w. Therefore, we can first scale the magnitude of w so that the denominator satisfies the constraint $w^{T}C_{j}w=1$.

$$\arg\;\underset{w}{\max}\;f\left(w\right)=\arg\;\underset{w}{\max}\;\frac{w^{T}C_{i}w}{1},$$

(A2) $$s.t.\;w^{T}C_{j}w=1.$$

Subsequently, we can utilize the method of Lagrange multipliers to address this optimization problem with constraint:

(A3) $$L\left(w,\lambda \right)=w^{T}C_{i}w-\lambda \left(w^{T}C_{j}w-1\right).$$

Taking the derivative of $L\left(w,\lambda \right)$ with respect to w and setting it to zero gives the following equation:

$$\nabla_{w}L=C_{i}w-\lambda C_{j}w=0$$

(A4) $$\Rightarrow C_{i}w=\lambda C_{j}w$$

Based on the definition of the generalized eigenvalue, λ is the generalized eigenvalue associated with matrices $C_{i}$ and $C_{j}$. By substituting $C_{i}w=\lambda C_{j}w$ into the definition of $f\left(w\right)$, we obtain the following:

(A5) $$f\left(w\right)=\frac{w^{T}C_{i}w}{w^{T}C_{j}w}=\frac{\lambda w^{T}C_{j}w}{w^{T}C_{j}w}=\lambda .$$

Thus, the maximum value of the objective function $f\left(w\right)$ is equal to the generalized eigenvalue associated with matrices $C_{i}$ and $C_{j}$. Furthermore, the global maximum of $f\left(w\right)$ is equal to the largest generalized eigenvalue.

## Appendix B

**Table A1:** Autoencoder architecture for clustering simulated EEG in the baseline Algorithm 5.

Module	Layer Type	Neurons	Activation Function	Loss Function
Input	–	21 (*n*(*n* + 1)/2)	–	–
Encoder	Fully connected	24	tanh	–
Encoder	Fully connected	12	tanh	–
Encoder	Fully connected	6	tanh	–
Latent	–	3	–	–
Decoder	Fully connected	6	tanh	–
Decoder	Fully connected	12	tanh	–
Decoder	Fully connected	24	tanh	–
Output	–	21	–	MSE

**Table A2:** Autoencoder architecture for clustering simulated EEG in SPADE algorithm.

Module	Layer Type	Neurons	Activation Function	Loss Function
Input	–	18 (3*n*)	–	–
Encoder	Fully connected	24	tanh	–
Encoder	Fully connected	12	tanh	–
Encoder	Fully connected	6	tanh	–
Latent	–	3	–	–
Decoder	Fully connected	6	tanh	–
Decoder	Fully connected	12	tanh	–
Decoder	Fully connected	24	tanh	–
Output	–	18	–	MSE

**Table A3:** Autoencoder architecture for clustering real EEG in SPADE algorithm.

Module	Layer Type	Neurons	Activation Function	Loss Function
Input	–	84 (3*n*)	–	–
Encoder	Fully connected	128	tanh	–
Encoder	Fully connected	64	tanh	–
Encoder	Fully connected	32	tanh	–
Latent	–	16	–	–
Decoder	Fully connected	32	tanh	–
Decoder	Fully connected	64	tanh	–
Decoder	Fully connected	128	tanh	–
Output	–	84	–	MSE

**Table A4:** Autoencoder architecture for clustering real EEG in the baseline Algorithm 5.

Module	Layer Type	Neurons	Activation Function	Loss Function
Input	–	406 (*n*(*n* + 1)/2)	–	–
Encoder	Fully connected	128	tanh	–
Encoder	Fully connected	64	tanh	–
Encoder	Fully connected	32	tanh	–
Latent	–	16	–	–
Decoder	Fully connected	32	tanh	–
Decoder	Fully connected	64	tanh	–
Decoder	Fully connected	128	tanh	–
Output	–	84	–	MSE
